# Thigh-length graduated compression stocking cannot increase blood velocity of the common femoral vein in patients awaiting total hip arthroplasty

**DOI:** 10.1186/s12891-022-05737-4

**Published:** 2022-08-11

**Authors:** Tao Jiang, Kai Song, Yao Yao, Zaikai Zhuang, Ying Shen, Xinhua Li, Zhihong Xu, Qing Jiang

**Affiliations:** 1 Nanjing Drum Tower Hospital Clinical College of Nanjing Medical University, 210008 Nanjing, People’s Republic of China; 2 State Key Laboratory of Pharmaceutical Biotechnology, Division of Sports Medicine and Adult Reconstructive Surgery, Department of Orthopedic Surgery, Nanjing Drum Tower Hospital, The Affiliated Hospital of Nanjing University Medical School, 321 Zhongshan Road, Nanjing, 210008 Jiangsu People’s Republic of China

**Keywords:** Graduated compression stockings, Blood velocity, Venous deformation, Deep vein thrombosis

## Abstract

**Objectives:**

Graduated compression stocking (GCS) is one of the mechanical prophylaxes commonly used for deep vein thrombosis (DVT). The present study was designed to observe the effects of graduated compression stockings on the vein deformation and hemodynamics of lower limbs in patients awaiting total hip arthroplasty (THA).

**Methods:**

The lower extremity veins of 22 patients awaiting THA were examined by ultrasound, when they rested in supine position with or without thigh-length GCS. The deformation parameters we measured included antero-posterior (AP) diameters, latero-medial (LM) diameters, and cross-sectional area (CSA) of great saphenous vein (GSV), posterior tibial vein (PTV), popliteal vein (PV), gastrocnemius vein (GV), and superficial femoral vein (SFV). We measured peak velocity and mean velocity of GSV, common femoral vein (CFV), junction of GSV and CFV to represent for hemodynamics of veins.

**Results:**

Significant compression was observed in almost all measured veins with the use of thigh-length GCS, while it was unable to significantly compress GSV in latero-medial diameter. The mean latero-medial diameter reductions for GSV, PTV, GV, PV and SFV were 19.4, 30.2, 43.2, 29.7 and 20.4%, respectively. GCS significantly compressed antero-posterior diameter of GSV, PTV, GV, PV and SFV by 43.4, 33.3, 42.1, 37.5, and 27.8%, respectively. The mean reduction of cross-section area was 44.8% for GSV, 49.6% for PTV, 60.0% for GV, 57.4% for PV, and 36.2% for FV. No significant changes were observed in the mean blood velocity of GSV, CFV, and junction. GCS was able to significantly reduce peak velocity of CFV (17.6 ± 5.6 cm/s to 16.1 ± 6.0 cm/s) and junction (23.3 ± 9.5 cm/s to 21.3 ± 9.7 cm/s), while it did not change the peak velocity of GSV.

**Conclusion:**

Thigh-length GCS is sufficient to compress lower extremity veins in patients awaiting THA in supine position with the greatest compression in GV, while it was unable to significantly increase blood velocity of common femoral vein or GSV. GCS may prevent DVT through more than simply increasing blood flow. Further studies are needed to determine the specific effects of GCS.

## Introduction

Patients undergoing orthopedic surgery, especially total hip arthroplasty (THA), total knee arthroplasty (TKA), and hip fracture surgery have been regarded as at the high-risk group for venous thromboembolism (VTE) [1]. The most common VTE developed in hospitalized patients is lower extremity deep vein thrombosis (DVT)[2].

DVT is characterized by abnormal blood coagulation within the deep veins resulting in clots and subsequent blockage of the veins. Virchow's law states that blood clots are caused by three factors, namely stasis, endothelial damage, and hypercoagulation. Patients typically manifest mild clinical symptoms such as edema, pain, and skin ulcers. Pulmonary embolism (PE) is a life-threatening complication of DVT. Without prophylaxis, DVT may lead to the PE at a rate of 28 to 41%, which may result in a 12% risk of 30-day mortality [3]. The post-thrombotic syndrome (PTS), which is a common sequela of DVT, is highly detrimental to the quality of life, as well as financially burden There is a 20 to 50% probability that patients who suffer from DVT will develop PTS, regardless of whether they are receiving antithrombotic therapies[4].

Both mechanical and pharmacological measures can be used to reduce the risk of DVT. The pharmacological approach primarily uses anticoagulants, which may increase the risk of postoperative hematoma and infection associated with hemorrhage. According to prior studies, even when low-molecular-weight heparin is used, 16% of patients develop venous thrombosis following THA [5]. The mechanical approach includes intermittent pneumatic compression (IPC), ankle pump, and graduated compression stockings (GCS). In light of the convenience and effectiveness of GCS, it has been widely recommended for prophylaxis against DVT [1]. In addition, it has been reported that GCS can effectively reduce the risk of DVT following orthopedic surgery [3]. Agu et al. [6] showed that wearing GCS resulted in a 57% reduction in the prevalence of DVT after THA.

While the mechanism of GCS preventing DVT is still not clear, some research has suggested that GCS may function by decreasing venous diameters in the lower extremities and increasing venous flow velocity [6, 7]. We have previously demonstrated that knee-length GCS can minish calf venous dilation but have no effect on the blood flow velocity of the femoral or popliteal veins in patients awaiting TKA in supine position [8]. However, peak velocity of vein is associated with the prophylaxis of DVT as Lachiewicz et al. [9] reported that higher peak velocity will lead to a lower rate of venous thrombosis. Besides, Keiler et al. [10] reported that femoral vein diameter increases with age and that there is strong correlation between CFV diameter and age in supine position.

The primary purpose of this study was to observe changes in venous deformation and hemodynamics of lower limbs of patients awaiting THA with or without wearing GCS in supine position. The secondary purpose of this study was to examine whether the age of patients affects the effect of GCS on blood velocity. We were interested in investigating the mechanism by which GCS reduces DVT morbidity.

## Materials and methods

Ethics approval for this study was granted by the Ethics Review Committee of hospital. Signed informed consent was received from All patients involved in the study.

Patients (≥ 18 years older and < 80 years) who were suffering from end-stage hip osteoarthritis, development dysplasia of the hip, osteonecrosis of femoral head, ankylosing spondylitis and rheumatoid arthritis were included in this study.

The exclusion criteria were as follows: obesity (body mass index > 30 kg/m^2^), underweight (body mass index < 18 kg/m^2^), thrombosis, chronic venous insufficiency, varicose veins, peripheral arterial disease, hematological disorders, Cardiovascular disorders, severe lower extremity deformities, taking anticoagulants recently, previously underwent lower extremity surgical procedures, or during pregnancy. Patients were also excluded if they were unable to cooperate with the ultrasound scan or there were not properly fitted stockings for them.

### Measurements of vein deformation and blood velocity

All measurements were performed in a quiet, temperature-controlled environment. Before measurements, the patients were asked to remove their shoes, pants, socks, and to lie in a supine position with both legs lying flat on the bed, breathing normally. Patients were instructed to rest for 15 min to allow hemodynamic status to stabilize.

We measured the antero-posterior (AP) diameters, latero-medial (LM) diameters, and cross-sectional area (CSA) of the following veins of the lower extremity that required THA. The great saphenous vein (GSV) and posterior tibial vein (PTV) was scanned at the midpoint of the calf. The popliteal vein (PV) was scanned at the popliteal fossa. The gastrocnemius vein (GV) was scanned at the position below the knee joint space. The superficial femoral vein (SFV) was scanned at the junction of the middle and lower third of the thigh.

The peak blood velocity and mean blood velocity were assessed at the following three positions:(1). CFV point: proximal part of the junction of the SFV and deep femoral vein (DFV). (2). GSV point: distal part of the junction of the GSV and CFV. (3). Junction point: proximal part of the junction of the GSV and CFV.

Marks were made on the skin with non-water-soluble black marker pens after baseline measurements were made to ensure that the subsequent scanning was at the same anatomical location.

To ensure that all data measured is accurate, patients were instructed to lie for an additional 15 min after we helped them put on the GCS. After the application of large amounts of gel on the marking sites, the vein can be imaged clearly with ultrasound. Then, we performed the second set of measurements on the patients.

In this study, all the deformation parameters were measured below the elastic band of the GCS over the upper thigh, and all the velocity parameters were measured above the band. When the transducer is pressed against the skin with additional pressure, modifications in parameters will occur, and this should be aware of.

**Fig. 1:**
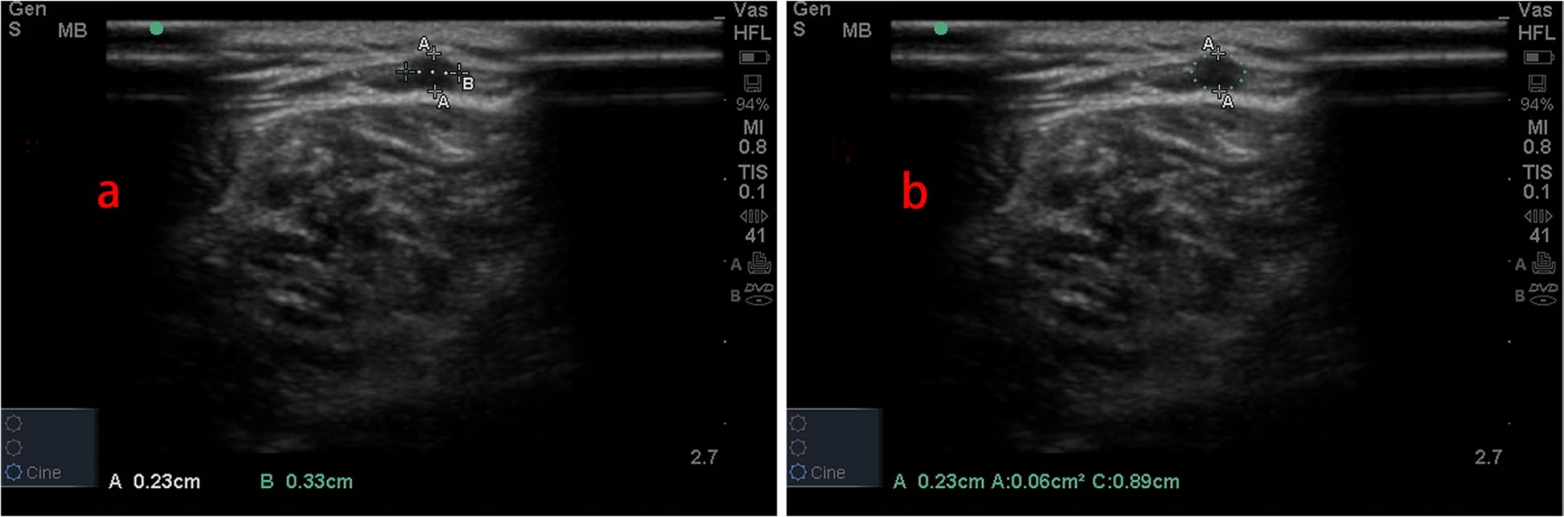
**a** Line A represents the antero-posterior diameters and line B represents the latero-medial diameters. **b** The cross-sectional area of vein

**Fig. 2:**
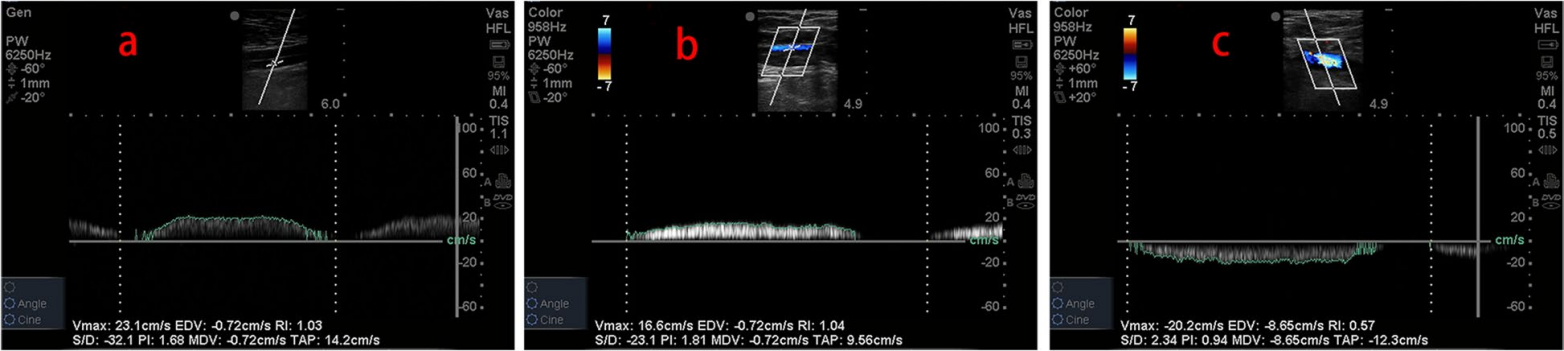
The Doppler sample volume cursor was positioned at the center of the venous lumen, and peak blood velocity and mean blood velocity were measured. **a** CFV point (proximal part of the junction of the superficial femoral vein and deep femoral vein). **b** GSV point (distal part of the junction of the GSV and CFV). **c** junction point (proximal part of the junction of the GSV and CFV)

All ultrasonic assessments were performed by a skilled physician, using a Sonosite M-Turbo ultrasound system. First, the transducer was placed on the horizontal axis of the vein. We froze images for assessments of AP diameters, LM diameters, and CSA when the transducer located the ideal positions (Fig. 1). A slight flexion and rotation of the knee joint were required in order to scan the PV. In addition, all patients were in the same posture when PV was measured to avoid deviations caused by posture or additional pressure. Peak velocity and mean velocity were obtained by placing the transducer longitudinally on the vein. The Doppler sample volume cursor was positioned in the center of the venous lumen and the Doppler wave was scanned three times separately (Fig. 2). Mean value was calculated by averaging three measurements.

### Graduated compression stockings

Thigh-length anti-embolism stockings (Perfectleg, Haoshide Medical Supplies Pty Ltd, China) were used in this study. According to product instructions, the appropriate size of the stockings was selected by measuring the ankle circumference, calf circumference (10 cm below the lower margin of the patella) and upper thigh circumference. The stockings were knitted from a blend of lycra and nylon threads and are capable of applying 16-22mmhg (class 1 compression) to patients' lower extremities.

### Statistical analysis

All figures were analyzed by SPSS (v.26, IBM Corp., Armonk, NY, USA). Results were presented as means ± standard deviations (SD). Wilcoxon rank-sum test was applied to analyze these results, with a significance threshold setting at *P* < 0.05.

## Results

In total, 22 patients were involved in the study. The characteristics of all patients were listed in Table 1.

**Table 1:** Patients ‘characteristics

Characteristic	Value
Age (years)	53.6 (35–74)
Sex (male/female)	9/13
Size of GSC (S/M/L)	14/5/3
Body mass index (Kg/m^2^)	24.0 (20.2–29.5)
Thigh circumference (cm)	48.8 (43.5–60.1)
Calf circumference (cm)	33.3 (29.0–39.5)
Ankle circumference (cm)	21.1 (18.5–26.5)

Values are means with ranges

The changes of LM diameters of veins in this study are shown in Table 2. GCS exhibited the most compression on GV from 4.4 ± 1.4 mm to 2.5 ± 1.7 mm(*P* < 0.01). There was no significant reduction observed in GSV (3.1 ± 1.1 mm vs 2.6 ± 1.1 mm, *P* = 0.07). GSV also exhibited significant reductions in PTV (4.3 ± 1.4 mm vs 3.0 ± 1.8, *P* < 0.01), PV (7.4 ± 1.5 mm vs 5.2 ± 1.9 mm, *P* < 0.01), and SFV (9.8 ± 2.1 mm vs 7.3 ± 3.2 mm, *P* < 0.01). GSV, PTV, GV, PV, and SFV had mean LM reductions of 19.4, 30.2, 43.2, 29.7, and 20.4%, respectively.

**Table 2:** The deformation of latero-medial diameters

LM (mm)	Without GCS	With GCS	*P*-value	Reduction%
GSV	3.1 ± 1.1	2.5 ± 1.7	0.07	19.4%
PTV	4.3 ± 1.4	3.0 + 1.8	< 0.01	30.2%
GV	4.4 ± 1.4	2.5 ± 1.7	< 0.01	43.2%
PV	7.4 ± 1.5	5.2 ± 1.9	< 0.01	29.7%
SFV	9.8 ± 2.1	7.8 ± 3.2	< 0.01	20.4%

Values are mean with SD

*GSV* Great saphenous vein, *PTV* posterior tibial vein, *GV*: Gastrocnemius vein, *PV* Popliteal vein, *SFV* Superficial femoral vein

GCS-related reductions in AP diameters of veins are shown in Table 3. GCS showed the most compression in GSV (2.3 ± 0.5 vs 1.3 ± 0.7, *P* < 0.01) and the least compression in SFV (7.2 ± 1.5 vs 5.2 ± 2.4, *P* < 0.01). GCS also decreased the AP diameter of GV from 6.4 ± 1.2 mm to 4.0 ± 1.7 mm(*P* < 0.01). There were additional reductions with the use of GSC in PTV (3.3 ± 1.1 mm vs 2.2 ± 0.9 mm, *P* < 0.01) and PV (6.4 ± 1.2 mm vs 4.0 ± 1.7 mm, *P* < 0.01). The mean reduction of AP diameters for GSV, PTV, GV, PV, SFV were 43.4%,33.3%,42.1%,37.5%,27.8%, separately.

**Table 3:** The deformation of antero-posterior diameters

AP (mm)	Without GCS	With GCS	*P*-value	Reduction%
GSV	2.3 ± 0.5	1.3 ± 0.7	< 0.01	43.4%
PTV	3.3 ± 1.0	2.2 ± 0.9	< 0.01	33.3%
GV	3.8 ± 1.1	2.2 ± 1.5	< 0.01	42.1%
PV	6.4 ± 1.2	4.0 ± 1.7	< 0.01	37.5%
SFV	7.2 ± 1.5	5.2 ± 2.4	< 0.01	27.8%

Values are mean with SD

*GSV* Great saphenous vein, *PTV* Posterior tibial vein, *GV* Gastrocnemius vein, *PV*: Popliteal vein, *SFV* Superficial femoral vein

Cross-sectional area for all veins measured are listed in Table 4. The most significant GSC-related compression in CSA was observed in GV (14.0 ± 9.3mm^2^ vs 5.6 ± 5.1 mm^2^, *P* < 0.01). GSC application was associated with a reduction in GSV from 5.8 ± 3.4 mm^2^ to 3.2 ± 3.1 mm^2^ (*P* < 0.01) and in PTV from 11.5 ± 6.8 mm^2^ to 5.8 ± 6.5 mm^2^ (*P* < 0.01). PV also showed GSC-related significant reduction in CSA from 39.7 ± 14.3 mm^2^ to 16.9 ± 11.0 mm^2^ (*P* < 0.01). The use of GCS additionally resulted in a reduction in SFV from 53.6 ± 22.2 mm^2^ to 34.2 ± 22.0 mm^2^ (*P* < 0.01). The mean reduction of CSA for GSV, PTV, GV, PV, SFV were 44.8, 49.6, 60.0, 57,4 and 36.2%, separately.

**Table 4:** The deformation of Cross-sectional area with or without GCS

CSA (mm^2^)	Without GCS	With GCS	*P*-value	Reduction%
GSV	5.8 ± 3.4	3.2 ± 3.1	< 0.01	44.8%
PTV	11.5 ± 6.8	5.8 ± 6.5	< 0.01	49.6%
GV	14.0 ± 9.3	5.6 ± 5.1	< 0.01	60.0%
PV	39.7 ± 14.3	16.9 ± 11.0	< 0.01	57.4%
SFV	53.6 ± 22.2	34.2 ± 22.0	< 0.01	36.2%

Values are mean with SD

*GSV* Great saphenous vein, *PTV* Posterior tibial vein, *GV* Ggastrocnemius vein, *PV* Popliteal vein, *SFV* Superficial femoral vein

Peak blood velocity and mean blood velocity of veins measured in the present study are shown in Table 5. GSC significantly reduced peak velocity in CFV (17.6 ± 5.6 cm/s to 16.1 ± 6.0 cm/s, *P* < 0.05) and junction (23.3 ± 9.5 cm/s to 21.3 ± 9.7 cm/s, *P* < 0.05) while it had no significant effects in GSV (1.2 ± 3.9 vs 11.6 ± 6.0 cm/s, *P* = 0.93). There were no significant changes in mean blood velocity in GSV (6.7 ± 3.1 cm/s vs 6.9 ± 3.7 cm/s, *P* = 0.96), CFV (9.5 ± 3.5 cm/s vs 8.9 ± 3.5 cm/s, *P* = 0.18) and junction (13.8 ± 6.5 cm/s vs 12.8 ± 6.2 cm/s, *P* = 0.06).

**Table 5:** Blood velocity of veins measured with or without GSC

Velocity (cm/s)	Without GCS	With GCS	*P*-value	Alteration%
GSV peak velocity	11.2 ± 3.9	11.6 ± 6.0	0.93	+ 3.6%
CFV peak velocity	17.6 ± 5.6	16.1 ± 6.0	< 0.05	-8.5%
Junction peak velocity	23.3 ± 9.5	21.3 ± 9.7	< 0.05	-8.6%
GSV mean velocity	6.7 ± 3.1	6.9 ± 3.7	0.96	+ 3.0%
CFV mean velocity	9.5 ± 3.5	8.9 ± 3.5	0.18	-6.3%
Junction mean velocity	13.8 ± 6.5	12.8 ± 6.2	0.06	-7.2%

Values are mean with SD

*GSV* Great saphenous vein, *CFV* Common femoral vein, *Junction* the junction of GSV and CFV

We divided the patients into two groups according to median age (55): elderly group (≥ 55 years old, *n* = 11) and young group (< 55 years old, *n* = 11). The blood velocity analysis of the two groups is presented in Table 6 and Table 7. In elderly group, there was no significant changes in peak blood velocity in GSV (10.1 ± 3.9 cm/s vs 12.2 ± 8.1 cm/s, *P* = 0.39), CFV (16.9 ± 6.4 cm/s vs 14.9 ± 5.9 cm/s, *P* = 0.08) and junction (23.9 ± 11.7 cm/s vs 23.4 ± 12.1 cm/s, *P* = 0.42). Meanwhile, GCS had no effects in mean blood velocity in GSV (5.9 ± 2.4 cm/s vs 6.9 ± 4.4 cm/s, *P* = 0.48), CFV (9.2 ± 3.5 cm/s vs 8.4 ± 3.4 cm/s, *P* = 0.08) and junction (14.2 ± 7.2 cm/s vs 13.8 ± 7.4 cm/s, *P* = 0.53). In young group, GCS significantly reduced peak velocity in junction (22.7 ± 7.2 cm/s vs 19.3 ± 6.5 cm/s, *P* < 0.05) while it had no effects in GSV (12.2 ± 3.8 cm/s vs 10.9 ± 3.2 cm/s, *P* = 0.27) and CFV (18.2 ± 5.0 cm/s vs 17.3 ± 6.1, *P* = 0.33). Besides, GCS couldn’t change mean velocity in GSV (7.5 ± 3.6 cm/s vs 6.8 ± 2.9 cm/s, *P* = 0.37), CFV (9.9 ± 3.6 cm/s vs 9.5 ± 3.6 cm/s, *P* = 0.66) and junction (13.4 ± 6.1 cm/s vs 11.7 ± 5.0 cm/s, *P* = 0.05).

**Table 6:** The peak blood velocity of veins measured in elderly group (≥ 55 years old, *n* = 11)

Velocity(cm/s)	Without GCS	With GCS	*P*-value	Alteration%
GSV peak velocity	10.1 ± 3.9	12.2 ± 8.1	0.39	+ 20.8%
CFV peak velocity	16.9 ± 6.4	14.9 ± 5.9	0.08	-11.8%
Junction peak velocity	23.9 ± 11.7	23.4 ± 12.1	0.42	-2.1%
GSV mean velocity	5.9 ± 2.4	6.9 ± 4.4	0.48	+ 16.9%
CFV mean velocity	9.2 ± 3.5	8.4 ± 3.4	0.08	-8.7%
Junction mean velocity	14.2 ± 7.2	13.8 ± 7.4	0.53	-2.8%

Values are mean with SD

*GSV* Great saphenous vein, *CFV* Common femoral vein, *Junction* the junction of GSV and CFV

**Table 7:** The blood velocity of veins measured in young group (< 55 years old, *n* = 11)

Velocity(cm/s)	Without GCS	With GCS	*P*-value	Alteration%
GSV peak velocity	12.2 ± 3.8	10.9 ± 3.2	0.27	-10.7%
CFV peak velocity	18.2 ± 5.0	17.3 ± 6.1	0.33	-4.9%
Junction peak velocity	22.7 ± 7.2	19.3 ± 6.5	< 0.05	-15.0%
GSV mean velocity	7.5 ± 3.6	6.8 ± 2.9	0.37	-9.3%
CFV mean velocity	9.9 ± 3.6	9.5 ± 3.6	0.66	-4.0%
Junction mean velocity	13.4 ± 6.1	11.7 ± 5.0	0.05	-12.7%

Values are mean with SD

*GSV* Great saphenous vein, *CFV* Common femoral vein, *Junction* the junction of GSV and CFV

## Discussion

This study indicated that fitted thigh-length graduated compression stockings could significantly compress almost all measured lower extremity veins in patients awaiting THA in a supine position, including GSV, PTV, GV, PV, and SFV. GCS couldn’t increase blood velocity of GSV, CFV and junction of CFV and GSV. It even reduced the peak velocity of CFV and junction. Furthermore, when patients were divided into two groups based on their age, GCS reduced the peak velocity of junction in the young group (< 55 years old).

In this study, we observed that the compression effects are most exhibited in GV with the AP, LM, CSA reduced 42.1%, 43.2%, 60.0% respectively, followed by PV. According to the results, GCS could lead to a significant reduction in diameter and CSA of lower extremity veins, which is consist with prior studies. Jeanneret et al. [11] concluded that thigh-length GCS were capable enough to decrease diameters of calf muscle veins in the prone position with a reduction ranging from 43.6 to 52.4% in GV. Coleridge Smith et al. [12] determined that GV diameter was reduced by 48% (2.6 mm to 1.6 mm) during surgical operations in stocking groups, while GV diameter increased by 19% (2.6 mm to 2.9 mm) in control groups.

According to the results, GSV showed a less pronounced compression with a 44.8% reduction in CSA, which is consistent with other previous studies. Partsch et al. [13] showed a more effective compression in the deep veins than superficial veins in prone position when wearing class 1 GCS (22 mmHg). Downie et al. [14] analyzed the deformation of 8 healthy volunteers’ lower leg veins through magnetic resonance imaging in the prone position. They indicated a 64% reduction in CSA of deep veins compare to 39% in superficial veins. This phenomenon can be explained by intramuscular pressure (IMP). A GCS of 22 mmHg can exert an additional 11 mmHg IMP on deep veins, whereas this pressure exerted by the subcutaneous layer is almost zero [15].

From the results, we observed that the LM diameter of almost all veins was less compressed when compared to AP diameter. Downie et al. [14] found that the cross-sections of deep veins are either orbicular or elliptical, whereas those of GSV are elliptical without compression. Their cross-sections were altered to elliptic after wearing GCS. This may explain the variance of deformation.

The reduction of diameters had been considered as a prerequisite of compression therapy [13], indicating that deformation of vein diameters is crucial to venous function. As a result of applying external pressure, GCS can ameliorate blood stasis and vascular endothelial tears [16, 17]. When blood stasis is present, vascular endothelial tears can contribute to the development of DVT by bringing thrombotic subendothelial collagen in contact with activated platelets, coagulation factors, or other thrombogenic factors [18].

As we anticipated, GCS could not significantly alter the mean blood velocity of the veins. We expressed more concern on peak velocity rather than mean velocity, as mean velocity is insensitive to moment-to-moment changes of velocity [19] while peak velocity is more accurate and reproducible [20]. Our results showed that GCS was able to significantly slow down the peak velocity of CFV and junction of CFV and GSV. This is varied from previous studies. Stein et al. [21] found no significant changes in blood velocity of femoral vein and popliteal vein of 26 hospitalized patients in supine position whether they had vein insufficiency or not. Keith et al. [19] compared the effect of using thigh-length and IPC boots individually or simultaneously in peak velocity of SFV. They revealed that GCS could not significantly increase the blood peak velocity. Furthermore, the use of GCS had no additional augmentation on peak velocity based on IPC boots. Norgren et al. [22] determined that GCS had no significant augmentation effects on peak velocity in the femoral vein of gravida during third-trimester pregnancy when they lay supine on the bed. However, different conclusions are drawn by others. Jamieson et al. [23] and Sigel et al. [24] indicated that GCS was sufficient accelerate femoral vein blood velocity in prone position,with a 38% augmentation in mean velocity mentioned in Sigel’s study.

The following are the reasons why we chosen to measure blood velocity in three points different from previous experiments. Our previous work had shown that keen-length GCS were unable to increase blood velocity in the femoral or popliteal vein in supine position[8]. Additionally, Lachiewicz et al. [9] determined that higher blood peak velocity above and below the junction of the GSV and CFV is associated with a lower prevalence of thrombosis.

When divided patients into two groups, we observed that GCS was sufficient to decrease the peak velocity of junction in the young group while it had no effect on other measured velocity values. Although no statistically significant difference was found, GCS decreased peak velocity (*P* = 0.08) and mean velocity (*P* = 0.08) in CFV. Considering the small number of patients studied, we may observe statistical significance once we conduct further study. The presence of marked venous stasis after THA would result in DVT [10]. The use of GCS should be combined with exercise in contrast to remaining stationary.

There are several limitations in the present study. First the sample size of our trial was small. Second, we only focused on preoperative patients because the measurements of postoperative patients would be disrupted by more factors. It is important to repeat measurements in postoperative patients in future studies. Third, several intravascular factors were disregarded, such as blood volume and vessel filling, which may have impacted the results of our study. Moreover, future studies should take postures and GCS pressure class into account.

## Conclusion

In summary, our results determined that thigh-length GCS was able to significantly compress the lower extremity vein of patients awaiting THA in supine position, with the greatest compression in the GV. GCS could not increase blood velocity of common femoral vein or GSV. It is possible that GCS may not achieve prevention effects only by increasing venous blood velocity. Further research is required to determine the specific effects of GCS.

## Data Availability

The datasets used and analyzed during the current study are available from the corresponding author on reasonable request.

## Abbreviations

GCS: Graduated compression stocking
DVT: Deep vein thrombosis
THA: Total hip arthroplasty
AP: Antero-posterior
LM: Latero-medial
CSA: Cross-sectional area
GSC: Great saphenous vein
PTV: Posterior tibial vein
PV: Popliteal vein
GV: Gastrocnemius vein
SFV: Superficial femoral vein
CFV: Common femoral vein
TKA: Total knee arthroplasty
VTE: Venous thromboembolism
PE: Pulmonary embolism
PTS: Post-thrombotic syndrome
IPC: Intermittent pneumatic compression
IMP: Intramuscular pressure
